# Effects of a 50% versus 100% pre-prandial insulin bolus reduction to improve glycemic safety during postprandial continuous and intermittent exercise in adults with type 1 diabetes treated with multiple daily injections

**DOI:** 10.3389/fendo.2026.1757429

**Published:** 2026-04-14

**Authors:** Warda Lahouel, Mouna Mnif, Mohamed Amine Bouzid, Faten Hadj Kacem, Elsa Heyman, Rémi Rabasa-Lhoret, Hamdi Frikha, Dhouha Zouari, Elodie Lespagnol, Monia Rekik, Mouna Turki, Sémah Tagougui

**Affiliations:** 1 Research Laboratory: Education, Motor Skills, Sports, and Health, EM2S, LR19JS01, University of Sfax, Sfax, Tunisia; 2 Higher Institute of Sports and Physical Education of Sfax, University of Sfax, Sfax, Tunisia; 3 Endocrinology Department, Hedi Chaker University Hospital, Sfax, Tunisia; 4 Univ. Lille, Univ. Artois, Univ. Littoral Côte d’Opale, ULR 7369 - URePSSS - Multidisciplinary Research Unit for Sport, Health and Society, Lille, France; 5 Institut Universitaire de France, Paris, France; 6 Montreal Clinical Research Institute, Montreal, QC, Canada; 7 Department of Nutrition, Faculty of Medicine, University of Montreal, Montreal, QC, Canada; 8 Division of Endocrinology, Montreal Diabetes Research Center, Montreal, QC, Canada; 9 Biochemistry Laboratory, Habib Bourguiba University Hospital, University of Sfax, Sfax, Tunisia; 10 Université Laval - Department of Operations and Decision Systems, Faculty of Business Administration, Québec, QC, Canada

**Keywords:** continuous exercise, insulin bolus, intermittent exercise, postprandial exercise, type 1 diabetes

## Abstract

**Objective:**

This study aimed to evaluate the effect of a 50% reduction in preprandial bolus insulin (50%-B) on plasma glucose (PG) responses during postprandial exercise of continuous moderate intensity (CONT) and intermittent high intensity (INT) in individuals with type 1 diabetes (T1D).

**Methods:**

Sixteen adults with T1D (31% male), treated with multiple daily insulin injections (MDI), participated in a randomized crossover study comprising four experimental conditions, separated by a washout period of at least 48 hours. Participants performed two 30-minute, preceded by a 3-minute warm-up without weights:

• CONT: continuous cycling at 60% of maximal aerobic power (MAP).

• INT: 2-minute intervals alternating between 40% and 80% of MAP, repeated for 7 intervals, with the last interval adjusted so that the total exercise time is exactly 30 minutes.

Each exercise modality was performed under two insulin conditions: a full preprandial bolus (100%-B) and a 50% reduction (50%-B). Plasma glucose, insulin, and cortisol were measured before, during, and after exercise. Linear mixed models were used to analyze temporal changes and condition effects.

**Results:**

Blood glucose decreased significantly over time for both exercise types (p < 0.001). During CONT, the decline in PG was similar between doses (Δ100%-B: –3.01 ± 2.96 vs. Δ50%-B: –2.82 ± 2.28 mmol/L; p = 0.18), However, the nadir PG was higher with 50%-B compared to 100%-B (8.59 ± 4.07 vs. 5.69 ± 3.06 mmol/L, respectively; β = +2.91 mmol/L; p = 0.026), and hypoglycemia was less frequent (2 vs. 18 episodes; p = 0.028). During INT, PG decreased less with 50%-B than with 100%-B (Δ: –2.03 ± 1.63 vs. –3.62 ± 2.76 mmol/L; p = 0.022), with no hypoglycemic episodes under 50%-B compared to six with 100%-B. Mean PG remained higher with 50%-B across both exercise types (p < 0.01). Plasma insulin decreased over time (p = 0.038) regardless of bolus condition, while cortisol increased more during INT with 100%-B than with 50%-B (p = 0.02).

**Conclusions:**

Reducing the preprandial bolus insulin by 50% effectively attenuates exercise-induced declines in plasma glucose and substantially reduces hypoglycemia risk, particularly during intermittent high-intensity exercise. These results emphasize the clinical relevance of personalized insulin adjustments to enhance metabolic safety during exercise in individuals with T1D.

## 1 Introduction

Regular physical activity (PA) has become an essential part of improving the well-being of people with type 1 diabetes (T1D) (1). It offers a range of benefits, including improved cardiorespiratory fitness, reduced insulin requirements, and an overall improvement in quality of life (2–4).

However, the prevalence of hypoglycemia during PA remains a significant barrier for people with T1D (5, 6). This increased risk results from the difficulty people with T1D have in adjusting their insulin doses during exercise, unlike people without diabetes (7). Despite the widespread use of insulin pumps, many patients rely on multiple daily injections (MDI) for the management of their diabetes (8), particularly in low- and middle-income countries where access to advanced diabetes treatment technologies remains limited (9, 10). In people living with T1D, exercise-induced hypoglycemia frequently occurs when activity is performed in the postprandial period, due to the presence of fast-acting insulin in the circulation (11). Several studies have shown that reducing the prandial bolus in anticipation is a strategy than can limit the reducion in PG during postprandial exercise, particularly during continuous aerobic exercise (12). Recent recommendations also emphasize the importance of taking into account the prandial context when adjusting insulin doses around exercise in order to reduce the risk of hypoglycemia (13, 14). However, this approach does not completely prevent hypoglycemia, particularly during postprandial aerobic exercise (15), highlighting the need for additional preventive strategies. Among the various factors influencing exercise-related glycemic responses, the type, intensity, and timing of PA play a crucial role (12, 16). Continuous aerobic exercise (CONT), characterized by sustained moderate intensity, increases the risk of hypoglycemia in patients with T1D (15), whereas intermittent exercise (INT), which alternates between periods of moderate and intense intensity, generally leads to a smaller decrease in glycemia levels and a lower risk of hypoglycemia than moderate-intensity exercise (17–19).

Although reducing the prandial insulin bolus is widely used, its comparative impact depending on the type of postprandial exercise remains insufficiently documented, particularly in people treated with MDI. To our knowledge, no study has experimentally and systematically examined the effect of reducing the prandial bolus during INT postprandial exercise, nor directly compared its impact during CONT exercise in this context. This gap limits the development of practical recommendations adapted to the type of exercise practiced.

In this context, the primary objective of this study was to evaluate the impact of a 50% reduction in preprandial insulin bolus on postprandial glycemic responses during two exercise modalities (CONT vs. INT) in people with T1D using multiple daily injections (MDI). Secondary objectives were to compare the risk of hypoglycemia between the two exercise modalities. We have formulated the following hypothesis: (i) a 50% reduction in insulin bolus would decrease the risk of hypoglycemia compared to the full dose (100%), without preventing the expected glycemic decline during exercise; (ii) the effect of insulin reduction would be modulated by the exercise modality (CONT vs. INT); (iii) an interaction between insulin dose and exercise type would influence the glycemic safety profile.

## 2 Methods

### 2.1 Population

16 participants (5 men and 11 women) were recruited for the study. Inclusion criteria included individuals aged ≥18 years or older, with diabetes for at least one year (no maximum duration was set as an inclusion criteria), using multiple daily injections (MDI) with stable doses for at least 3 months prior to inclusion, and with a recent A1c result (within the last two months) ≤ 10% (86 mmol/mol). Exclusion criteria included clinically significant microvascular complications such as nephropathy (glomerular filtration rate < 40 mL/min), specific neuropathy (particularly gastroparesis), or proliferative retinopathy. Other exclusion criteria included recent macrovascular events within the last three months, anemia or abnormal blood test results, pregnancy, severe hypoglycemia within the last two weeks, any medical condition deemed by the investigator to interfere with the protocol, and failure to comply with the research team’s recommendations.

The study was conducted in the endocrinology and diabetes department of the University Hospital of Sfax. In addition, consumption of caffeinated beverages and other stimulants (e.g., nicotine, dietary supplements, energy drinks, etc.) was prohibited 24 hours prior to the procedure in order to minimize potential confounding factors. The study protocol was approved by the local clinical research ethics committee (CPP SUD No. 0426/2022) and was conducted in accordance with the Declaration of Helsinki. On April 5, 2023, all participants signed an informed consent form, and the trial was registered in the African Clinical Trials Registry (PACTR202304590853621).

#### 2.1.1 Admission visit

During a medical examination to assess inclusion and exclusion criteria, it was confirmed that individuals with T1D had no microvascular or macrovascular complications, nor a history of cardiovascular events, cerebrovascular insufficiency, or coronary insufficiency. Blood samples were collected to measure A1C levels. Height and weight were recorded, and body composition was assessed using bioelectrical impedance analysis (Tanita TBF300). To determine MAP, an incremental exercise test to exhaustion was performed on a Monark Ergomedic 874E exercise bike (Monark AB, Varberg, Sweden). After a 3-minute warm-up, the load was increased by 30 W every 2 minutes until voluntary exhaustion. MAP corresponded to the last power level fully completed. Heart rate was continuously recorded using a heart rate monitor (Polar^®^, Finland), and perceived exertion was assessed every 2 minutes using the Borg 6–20 scale (RPE) (20). Reaching maximum effort and voluntary exhaustion was defined as the inability to maintain the target cadence despite verbal encouragement, a high perceived exertion (Borg scale ≥ 19/20), and reaching at least 90% of the age-predicted maximum heart rate. MAP was defined as the last load completed. In addition, patients completed the International Physical Activity Questionnaire (IPAQ) to provide additional information on their level of PA (21, 22). During this first visit, patients were asked to indicate their usual insulin doses (rapid-acting analogue: Novorapid and long-acting analogue: Lantus or Levemir).

In addition, before each experimental session, dietary intake was standardized based on a structured, non-validated dietary survey conducted by a dietitian during the preliminary consultation. This survey, used in routine clinical practice in the endocrinology department.

### 2.2 Intervention visits

**Figure 1:**
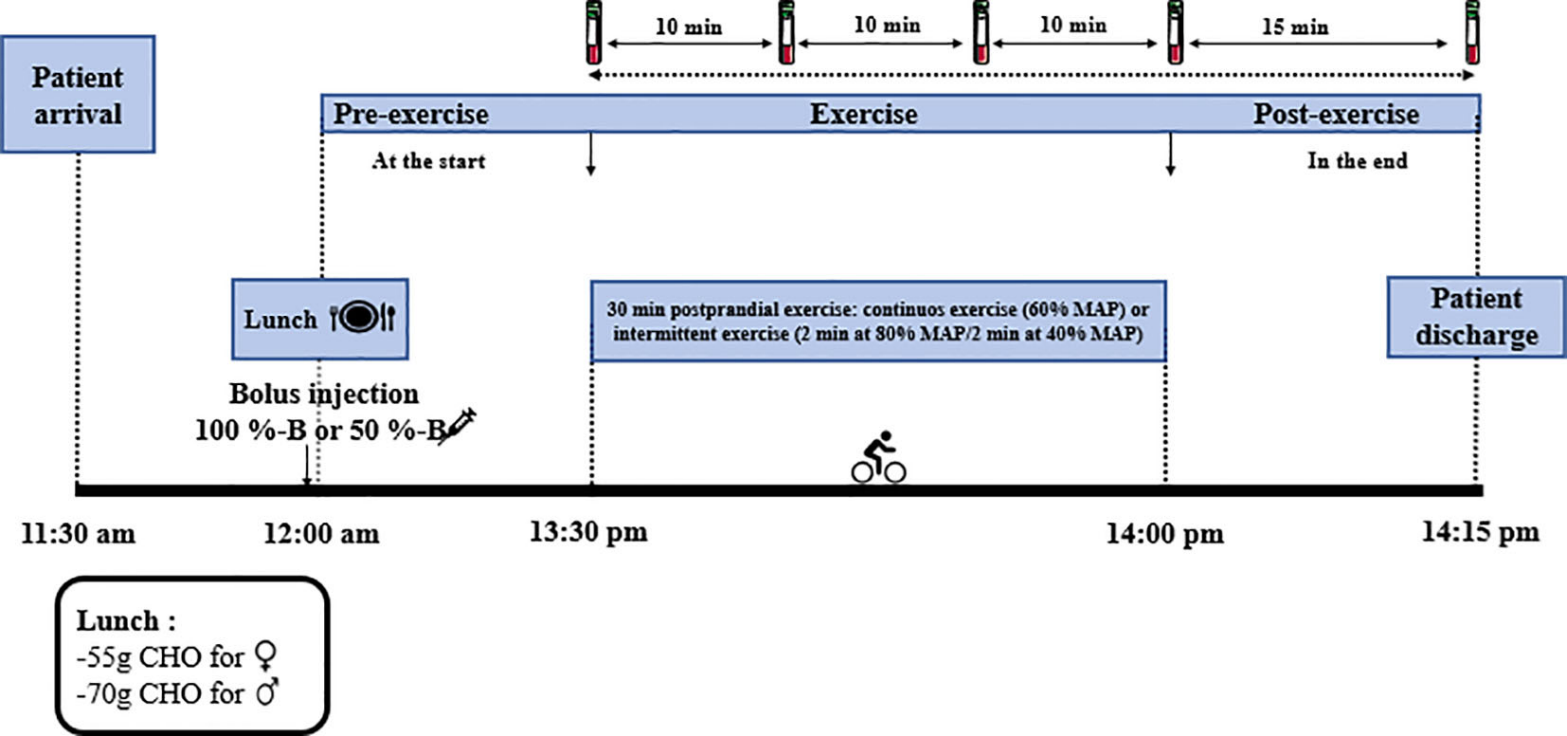
Description of the procedures used during the study.

The protocol of the intervention visits is presented in **Figure 1**. To minimize potential confounding factors, participants were asked about the time of their last meal and insulin dose before exercise and were instructed to avoid PA, except for daily walking, on the day before and the day of the interventions. Participants were instructed to consume a standardized lunch designed and supervised by a nutritionist to ensure comparable metabolic conditions for all visits. During each intervention session, participants consumed a standardized lunch, providing 55 g of carbohydrates for women and 70 g for men, with a constant protein/fat ratio. Meals were prepared using simple cooking methods (steaming or grilling) to ensure consistent postprandial absorption and minimize the influence of food composition on glycemic responses.

The preprandial insulin bolus was administered immediately before the meal, according to two randomized conditions: 50% of the usual bolus (50%-B) or the full dose (100%-B), during the four intervention visits. The reference dose corresponded to the dose usually used by each participant for that meal, calculated according to their personal insulin/carbohydrate ratio. As part of the experimental protocol, this dose was administered either at 100% or reduced by 50%. Preprandial blood glucose was not used to adjust the insulin dose in the study.

Participants ate lunch at 12 pm, and each exercise session began 90 minutes after the meal, in random order, to minimize the influence of circadian rhythm on blood glucose and hormonal responses. The visits were scheduled to avoid the menstrual period in order to limit the influence of hormonal fluctuations on glucose regulation (23). Participants completed four randomized visits, combining either a full bolus dose or a bolus dose reduced by 50%, and either CONT exercise or INT exercise. Each session consisted of 30 minutes of cycle ergometry at 60% of MAP for the CONT exercise, while the INT exercise consisted of alternating intervals of 2 minutes of cycling at 80% MAP and 2 minutes at 40% MAP, with the last interval adjusted to achieve a total duration of 30 minutes. A standardized 3-minute warm-up without load preceded each session. The experimental sessions were separated by a 48-hour interval. In order to verify that participants maintained the target intensity and that the relative load was comparable between them, perceived exertion (RPE, Borg scale 6–20) was assessed during each session, at different phases of the exercise. Throughout the exercise sessions, blood samples were taken intravenously at the start (90 minutes after the meal) (T1), 10 minutes after the start of exercise (T2), 20 minutes after the start of exercise (T3), at the end (T4), and 15 minutes after exercise (T5) to assess venous glycemia levels. Cortisol and insulin were measured at the start and end of exercise. An oral carbohydrate supplement (juice; 12 to 24 g of carbohydrates) was administered whenever the measured capillary blood glucose level fell below 70 mg/dL (4 mmol/L) (24), regardless of whether hypoglycemic symptoms were present. If the capillary glucose level did not return to a safe level (> 70 mg/dL), carbohydrate intake could be repeated until a safe level was reached.

### 2.3 Blood sampling

Blood volumes were adjusted according to the planned analyses. For the combined measurement of venous glucose, insulin, and cortisol (T1 and T4), approximately 3–5 mL of blood were collected per lithium heparin tube. For other time points where only venous glucose was measured, 2 mL were collected. These volumes comply with the recommendations of the Clinical & Laboratory Standards Institute (CLSI, GP41) regarding the blood/additive ratio, ensuring the validity of enzymatic hexokinase measurements and minimizing analytical interference due to underfilling or inappropriate ionic strength. Plasma glucose was measured using the hexokinase enzymatic method with a commercial kit from Roche Diagnostics (GmbH, Mannheim, Germany). Peripheral blood was collected in a lithium heparin tube. Samples were centrifuged for 15 minutes at 3,000 rpm. Plasma samples were immediately analyzed for PG content using the hexokinase reference enzymatic method, and an aliquot was stored at -80 °C for subsequent analysis of total insulin and cortisol using the ECLIA electrochemiluminescence immunoassay. HbA1c was measured in hemolyzed whole blood using the Tina-quant Hemoglobin A1c Gen.3 test (Roche Diagnostics, Germany) on the Roche/Hitachi cobas 6000 c501 analyzer. All samples were analyzed on the same analyzer with the same reagent kit to minimize analytical variability.

### 2.4 Sample size calculation

The sample size was calculated using G*Power (3.1.9.7) and, in the absence of precise data on the effect size for this population, a standardized effect size of F = 0.25 was used, considered a small effect size according to Cohen’s recommendation. Alpha values were set at 0.05 and power (1-Beta) at 0.80. Finally, 16 participants were deemed sufficient to reduce the risk of type II statistical error. Eighteen volunteers were initially included to account for any possibility of dropouts during the experiments. The experiment was ultimately conducted with 16 participants, as two dropouts were recorded, one for personal reasons and the other due to an illness unrelated to the study.

### 2.5 Statistical analysis

Mixed linear models were used to test differences between conditions (CONT 100%-B, CONT 50%-B, INT 100%-B, INT 50%-B) for all numeric outcome variables, with patient ID as a random effect and condition as a fixed effect. For the analysis of glycemia measured at different times, time was also included as a fixed effect in the model, along with its interaction with the experimental condition. The interaction term was then removed from the model if it was not significant. For the parameters Δ glycemia and nadir glycemia, the pre-exercise value was added as a covariate in the model. For significant categorical variables, *post-hoc* pairwise comparisons between conditions were performed using Bonferroni correction for multiple testing. P < 0.05 was considered statistically significant. All statistical analyses were performed in R version 4.2.1.

## 3 Results

A total of 16 adults with T1D using MDI were recruited. The baseline characteristics of the participants are summarized in **Table 1**.

**Table 1:** Characteristics of participants.

Characteristics	Mean ± SD	
Number (M/W) (n)	16 (5/11)	
Age (years)	26.19 ± 5.78	
Height(m)	1.66 ± 0.08	
BMI (kg/m^2^)	24.03 ± 4.03	
Lean body mass (%)	49.05 ± 7.39	
Body fat (kg)	16.84 ± 10.35	
Basic metabolism (kcal)	1557.23 ± 154.91	
Water mass (kg)	35.26 ± 4.54	
A1C mmol/mol (%)	74 ± 17.24 (8.92 ± 1.57)	
Duration of diabetes (years)	14.13 ± 6.22	
Total daily insulin dose per day (U kg^−1^)	Basal insulin	0.38 ± 0.21
	Bolus insulin	0.51 ± 0.26
MAP (Watt)	87.66 ± 26.98	
IPAQ (MET-minutes/week)	313.13 ± 150.63	

BMI, Body mass index; A1C, glycated hemoglobin; MAP, Maximal aerobic power; IPAQ, International Physical Activity Questionnaire.

**Figure 2:**
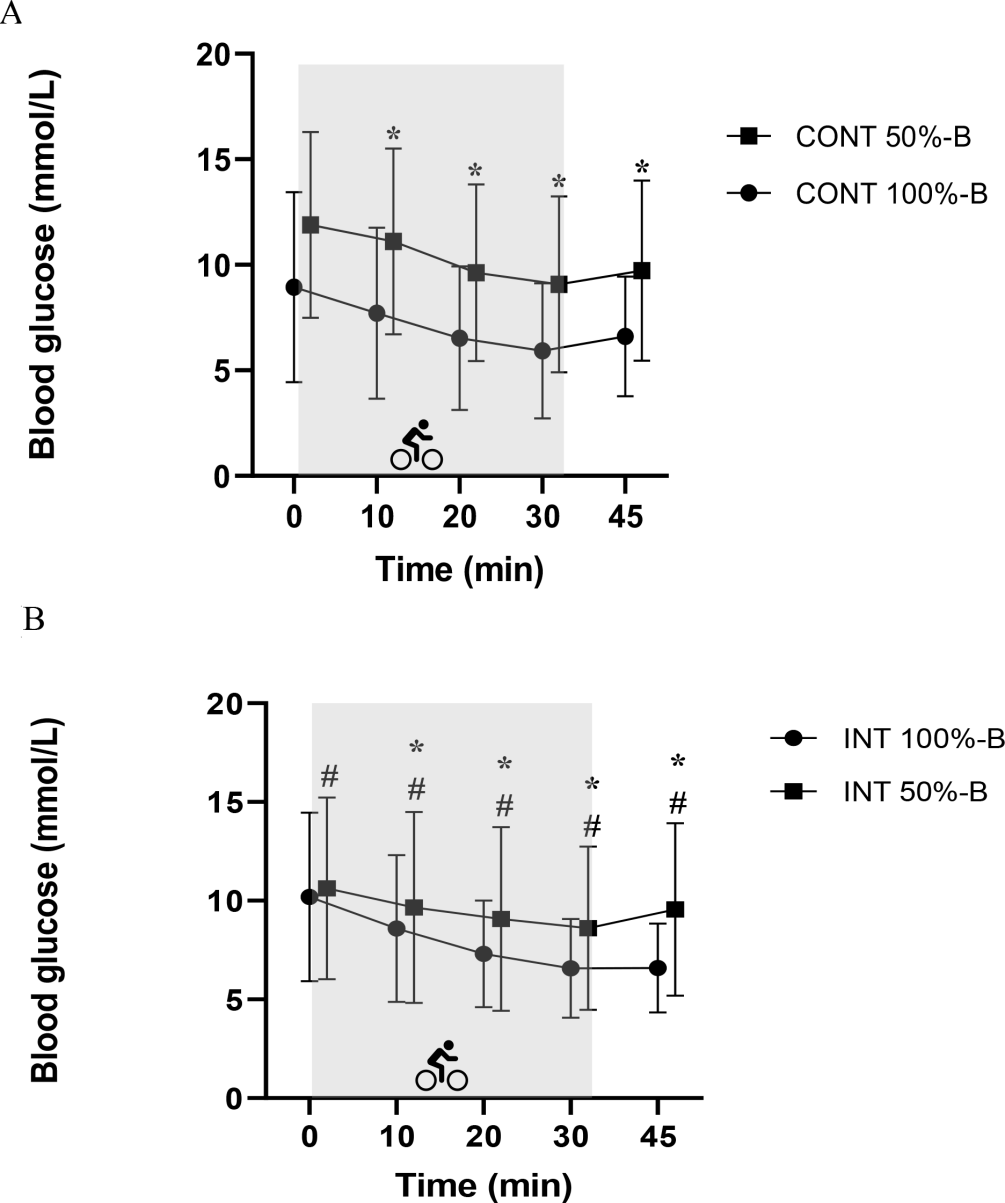
Change in blood glucose over time in both strategies during CONT exercise **(A)** and INT exercise **(B)**; 50% B – squares, 100% B – circles. The shaded area corresponds to the exercise session. Data are expressed as mean ± standard deviation. Main effects of the mixed model during the 30-minute exercise period: time and strategies. *Post hoc* analyses for the effect of time significantly different from that at rest *P < 0.05, #significant effect between the two strategies; p < 0.05.

Analysis using a linear mixed model showed that blood glucose levels decreased significantly over time for both types of exercise (p < 0.001) (**Figure 2**). Baseline blood glucose (T1) was an independent and significant predictor of blood glucose change during exercise. In other words, the higher the baseline blood glucose level, the smaller the decrease (β = –0.34 mmol/L; 95% CI [–0.45 to –0.22]; p < 0.001).

During CONT exercise, the decrease in blood glucose did not differ significantly between the two bolus doses (β = +1.12 mmol/L; 95% CI [-0.53 ― 2.78]; p = 0.176). In contrast, the blood glucose nadir was higher with 50%-B compared to 100%-B (8.59 ± 4.07 vs. 5.69 ± 3.06; respectively, β = +2.91 mmol/L; 95% CI [0.03 ― 5.78]; p = 0.026) and the incidence of hypoglycemia was lower (2 vs. 18 episodes; p = 0.028), indicating a safer metabolic profile.

For INT exercise, PG rates also decreased, but less markedly with 50%-B compared to 100%-B (2.03 ± 1.63 vs. -3.62 ± 2.76 mmol/, respectively; β = +1.688 mmol/L/min; 95% CI: 0.06 ― 3.32; p = 0.022). No episodes of hypoglycemia were observed with 50% B (0 episodes; CHO = 0 g) compared with six episodes with 100% B, requiring 24 g of CHO. In addition, mean venous blood glucose levels remained higher with 50% B (β = +1.53 mmol/L; 95% CI [0.2 ― 2.85]; p = 0.007), reflecting increased metabolic safety (**Table 2**).

**Table 2:** Glucose, Insulin, and Cortisol outcome during both types of exercise.

Outcome	CONT exercise			INT exercise			P condition
	100%-B	50%-B	P-value (comparisons *post-hoc* (Bonferroni)	100%-B	50%-B	P-value	
PG (mmol/L)	T1 = 8.94 ± 4.50 T4 = 5.93 ± 3.20***	T1 = 11.69 ± 4.40 T4 = 8.88 ± 4.17***	P < 0.0001	T1 = 10.19 ± 4.26 T4 = 6.57 ± 2.50***	T1 = 10.43 ± 4.60 T4 = 8.4 ± 4.14***	P = 0.007	P < 0.0001
ΔPG (mmol/L) from start to end of the exercise, controlling the baseline PG	-3.01 ± 2.96	-2.82 ± 2.28	P = 0.176	-3.62 ± 2.76	-2.03 ± 1.63	P = 0.022	P = 0.018
Nadir glucose (mmol/L) during exercise, controlling the baseline PG	5.69 ± 3.06	8.59 ± 4.07	P = 0.026	6.42 ± 2.58	8.19 ± 4.24	P = 0.238	P = 0.026
Insulin (pmol/L)	T1: 11.57 ± 15.62 T4: 10.79 ± 16.76*	T1: 8.94 ± 12.67 T4: 8.39 ± 11.16*	P = 0.180	T1: 10.84 ± 17.08 T4: 8.94 ± 13.27*	T1: 9.94 ± 13.32 T4: 6.42 ± 8.72*	P = 0.232	P = 0.279
Cortisol (ng/mL)	T1: 109.36 ± 59.35 T4: 107.44 ± 50.26	T1: 80.28 ± 20.14 T4: 85.27 ± 46.48	P = 0.079	T1: 99.46 ± 51.21 T4: 136.13 ± 79.40	T1: 91.87 ± 55.20 T4: 112.48 ± 73.16	P = 0.020	P = 0.0008
No. of hypoglycemia events, n	18	2	P = 0.028	6	0	-–	-–
Number of participants requiring treatment for hypoglycemia	6/16	1/16	P = 0.090	2/16	0/16	-–	-–
Total carbohydrate requirement (g)	168	24	P = 0.091	24	0	-–	-–

Data expressed as mean ± standard deviation (SD); PG, plasma glucose; T1, pre-exercise; T4, end of exercise. *P <0.05, ***P <0.001: T1 vs T4 (effect of time in the same condition); P-value (Bonferroni *post-hoc* comparisons): 100%-B vs 50%-B in the same type of exercise. P (condition): overall effect of the condition (comparison of the four conditions together).

For both types of exercise, no significant differences were observed between the two bolus doses at the end of exercise and after the 15-minute recovery period (β = +0.57 mmol/L/min; 95% CI [-0.98 ― 2.12]; p = 0.317). In addition, patients with T1D had significantly higher blood glucose levels at the start of, during, and after exercise with 50% B compared to 100% B during both types of exercise (CONT: β = +2.94 mmol/L; 95% CI [1.65 ― 4.24]; p < 0.0001; INT: β = +1.53 mmol/L; 95% CI [0.2 ― 2.85]; p = 0.007).

Cortisol analysis revealed a significant effect of condition (p = 0.0008), while the effect of time showed no significant difference (p = 0.071). *Post hoc* analysis showed a significant decrease in cortisol for INT exercise when the insulin bolus was reduced by 50% compared to the full bolus condition (β = −21.02 ng/mL; 95% CI [−41.69 − 0.35]; p = 0.02). For CONT exercise, no significant difference was observed between the two insulin doses (β = −17.12 ng/mL; 95% CI [−38.28 – 4.04]; p = 0.079) (**Table 2**). These results suggest that reducing the insulin bolus attenuates the exercise-induced corticotropic response during INT exercise.

Insulin concentrations decreased significantly over time (β = –1.71 pmol/L/min; 95% CI [–3.31; –0.11]; p = 0.038) (**Table 2**), while no significant effect of condition was observed (p = 0.279). These results suggest a decrease in insulin levels independent of exercise type and bolus reduction strategy.

## 4 Discussion

This study provides further evidence of the impact of a 50% reduction in preprandial insulin bolus (50%-B) in patients with type 1 diabetes (T1D) following multiple daily injections (MDI) and engaging in intermittent (INT) or continuous (CONT) exercise after meals. Our results show that the 50%-B strategy significantly attenuates the decline in plasma glucose (PG) during INT exercise, whereas no difference was observed for CONT exercise when comparing 50%-B to the full bolus dose (100%-B).

Although the decrease in PG did not differ significantly between the two insulin doses during CONT exercise, it is important to note that the 50%–B strategy was associated with a decrease in hypoglycemic episodes requiring carbohydrate intervention. These results are consistent with recent studies showing that preventive insulin adjustment improves glycemic control around moderate to high intensity (50-70% VO_2_max) CONT exercise for 30 to 60 minutes in patients with T1D (25, 26). This effect could be partly attributed to insulin-independent glucose uptake mechanisms, such as GLUT4 translocation triggered by muscle contraction (27). In addition, the glycemic nadir was significantly higher with the 50%-B strategy, reinforcing its potential role in preventing hypoglycemia. A notable finding was the higher pre-exercise plasma glucose (T1) observed in the CONT 50%-B condition compared with CONT 100%-B, a difference that was not present in the INT sessions. Despite the randomized crossover design, such variability likely reflects normal day-to- day postprandial glycemic fluctuations in people with T1D treated with MDI. Importantly, baseline glucose was included as a covariate in the statistical models for ΔPG and nadir glucose. Moreover, higher starting glucose levels were independently associated with a smaller exercise-induced decline, suggesting a regression-to-the-mean phenomenon. Therefore, the reduced hypoglycemia risk observed with the 50%-B strategy during CONT exercise cannot be solely attributed to higher pre-exercise glucose levels. Rather, it likely reflects lower circulating insulin concentrations at exercise onset, which attenuate glucose disposal during moderate continuous activity and improve glycemic safety.

During INT exercise, PG also decreased, but this decrease was less pronounced with 50%-B strategy. Despite this attenuation, no significant difference was observed at nadir during this type of exercise. These results could be explained by the alternation between intense efforts (10 intervals of 30 seconds each at 100–120 % VO_2_max) and 2 minutes of active recovery at 40–50 % VO_2_max, for a total duration of 30 minutes, which promotes more stable glucose kinetics (28). In addition, the increase in cortisol observed only in the 100% B condition reflects the body’s counter-regulatory response to a more rapid drop in glucose: cortisol acts to increase blood glucose levels and limit the risk of hypoglycemia. In contrast, with a bolus reduced to 50%, blood glucose decreases more gradually and steadily, thereby reducing the stimulus for cortisol release. These observations suggest that adjusting the preprandial insulin dose not only attenuates the glycemic decline during INT exercise, but also limits the hormonal stress response, reinforcing the glycemic safety profile. This highlights the clinical value of reducing the preprandial bolus for postprandial INT exercise in people with T1D treated with MDI (26). However, these results should be interpreted with caution, as the intensity of the exercise was not objectively confirmed by direct physiological measurements, which may introduce interindividual variability in metabolic load.

During both types of exercise, plasma glucose (PG) levels were higher before, during, and after exercise with the 50% bolus (50%-B) strategy compared to the full dose (100%-B). Pre-exercise plasma glucose (T1) was higher in the CONT 50%-B condition compared with CONT 100%-B. To account for this imbalance, baseline glucose was included as a covariate in the mixed models for ΔPG and nadir glucose. Moreover, higher T1 values were independently associated with a smaller exercise-induced decline (β = –0.34 mmol/L; p < 0.001). Therefore, the reduced hypoglycemia risk observed with the 50%-B strategy during CONT exercise cannot be solely attributed to differences in pre-exercise glucose levels. Interestingly, although baseline PG was elevated, previous studies have shown that higher pre-exercise glucose generally lead to larger decreases during prolonged aerobic exercise (~60 minutes) (29). In contrast, we observed that the 50%-B strategy helped limit excessive glucose decreases, suggesting that reducing the preprandial insulin bolus may stabilize glycemia during exercise, despite higher starting glucose levels. This discrepancy with prior findings can be explained by several mechanisms: (i) the lower insulin bolus limits rapid glucose uptake by muscles, (ii) it reduces the diffusion gradient between the blood and muscle when insulin sensitivity decreases, and (iii) it further restricts glucose penetration into muscles before exercise (26). These results highlight the importance of adjusting preprandial insulin doses based on baseline glucose levels, in line with ISPAD recommendations (30), to improve glycemic stability and minimize hypoglycemia risk during PA.

In addition, plasma insulin concentration decreased significantly over time, with no notable difference between conditions (type of exercise or bolus reduction). These results are consistent with the results of (31), who showed that exercise, regardless of type (CONT or INT), promotes a decrease in circulating insulin concentrations through increased clearance and improved glucose uptake by muscles (32, 33).

Future research should include other counterregulatory markers, such as catecholamines and glucagon, to better characterize hormonal responses to exercise in the context of varying insulin doses. To our knowledge, this study is the first to directly compare the effects of reducing preprandial insulin bolus during postprandial exercise according to two modalities (INT vs. CONT) in people with T1D on MDI therapy. The crossover design, standardized conditions, and direct comparison of exercise modalities are important methodological strengths. However, the robustness and clinical relevance of the results should be interpreted with caution due to limitations related to imperfect control of dietary intake and PA between visits. The use of biochemical markers such as plasma insulin and cortisol also provides relevant physiological insight. However, certain limitations must be taken into consideration. The relatively small sample size and gender imbalance limit the generalizability of the results and do not allow for subgroup analyses or the detection of small effects, despite the adequacy of the planned and actual sample sizes.

Furthermore, the study only focused on 30 minutes of exercise, which limits the extrapolation of the results to other durations of effort or situations involving prolonged PA. We did not measure free (bioactive) insulin or preprandial PG, and insulin doses were not adjusted for body weight, which may contribute to the interindividual variability observed. Finally, although necessary, controlled experimental conditions may limit the direct extrapolation of results to real-life situations.

Further studies are needed to explore the effect of different amplitudes of insulin bolus reduction at various times of the day and during prolonged or nocturnal recovery phases. The integration of data from continuous glucose monitoring and a more comprehensive assessment of counterregulatory hormones would provide a better understanding of physiological adaptations to exercise in the context of insulin adjustment. The exercise intensity was prescribed based on individual maximum aerobic power, without concomitant physiological measurements (VO_2_, heart rate, or energy expenditure). Although the RPE made it possible to verify adherence to the target intensity, this approach does not confirm strict equivalence of internal metabolic load between participants, which must be taken into account when interpreting metabolic results. Furthermore, the inclusion of participants with a wide variation in disease duration may have influenced metabolic responses to exercise, particularly through individual differences in insulin sensitivity, hormonal regulation, or glycogen stores. This heterogeneity should be taken into account when interpreting the results. The lack of formal validation of the dietary survey also constitutes a methodological limitation of the study. Finally, while experimental controls were necessary, they may limit direct generalization to real-world exercise conditions.

Further studies are needed to explore the effect of insulin bolus reductions at different times of the day and during prolonged recovery and nighttime periods. Integration of continuous glucose monitoring data and assessment of counterregulatory hormone dynamics will provide insight into physiological adaptations to exercise with modified insulin dosing.

In conclusion, our results suggest that a 50% reduction in preprandial insulin bolus could improve glycemic stability and reduce the risk of hypoglycemia during postprandial exercise, particularly in the context of INT exercise, in people with T1D on MDI. However, these observations need to be confirmed by larger studies conducted under real-life conditions before they can be generalized to clinical practice.

## Data Availability

The original contributions presented in the study are included in the article/supplementary material. Further inquiries can be directed to the corresponding author.
